# Quality of care transition, patient safety incidents, and patients’ health status: a structural equation model on the complexity of the discharge process

**DOI:** 10.1186/s12913-024-11047-3

**Published:** 2024-05-03

**Authors:** Matthias Marsall, Thorsten Hornung, Alexander Bäuerle, Matthias Weigl

**Affiliations:** 1 Institute for Patient Safety (IfPS), University Hospital Bonn, Bonn, Germany; 2 University Hospital Bonn, Bonn, Germany; 3 Clinic for Psychosomatic Medicine and Psychotherapy, LVR–University Hospital Essen, University of Duisburg-Essen, Essen, Germany; 4 Center for Translational Neuro- and Behavioral Sciences (C-TNBS), University of Duisburg-Essen, Essen, Germany

**Keywords:** Transitional safety, Care transitions measure, Structural equation model, Readmission, Medication safety, Discharge, Patient-reported experience measure, Patient-reported outcomes measures

## Abstract

**Background:**

The transition of patients between care contexts poses patient safety risks. Discharges to home from inpatient care can be associated with adverse patient outcomes. Quality in discharge processes is essential in ensuring safe transitions for patients. Current evidence relies on bivariate analyses and neglects contextual factors such as treatment and patient characteristics and the interactions of potential outcomes. This study aimed to investigate the associations between the quality and safety of the discharge process, patient safety incidents, and health-related outcomes after discharge, considering the treatments’ and patients’ contextual factors in one comprehensive model.

**Methods:**

Patients at least 18 years old and discharged home after at least three days of inpatient treatment received a self-report questionnaire. A total of *N* = 825 patients participated. The assessment contained items to assess the quality and safety of the discharge process from the patient’s perspective with the care transitions measure (CTM), a self-report on the incidence of unplanned readmissions and medication complications, health status, and sociodemographic and treatment-related characteristics. Statistical analyses included structural equation modeling (SEM) and additional analyses using logistic regressions.

**Results:**

Higher quality of care transition was related to a lower incidence of medication complications (B = -0.35, *p* < 0.01) and better health status (B = 0.74, *p* < 0.001), but not with lower incidence of readmissions (B = -0.01, *p* = 0.39). These effects were controlled for the influences of various sociodemographic and treatment-related characteristics in SEM. Additional analyses showed that these associations were only constant when all subscales of the CTM were included.

**Conclusions:**

Quality and safety in the discharge process are critical to safe patient transitions to home care. This study contributes to a better understanding of the complex discharge process by applying a model in which various contextual factors and interactions were considered. The findings revealed that high quality discharge processes are associated with a lower likelihood of patient safety incidents and better health status at home even, when sociodemographic and treatment-related characteristics are taken into account. This study supports the call for developing individualized, patient-centered discharge processes to strengthen patient safety in care transitions.

**Supplementary Information:**

The online version contains supplementary material available at 10.1186/s12913-024-11047-3.

## Background

Healthcare transitions, such as patient transfers from hospital to home or from one care setting to another, are high-risk processes in terms of patient safety [1–3]. The World Health Organization (WHO) highlighted the importance of safe care transitions in its Patient Safety Action Plan 2021–2030 as one major goal of eliminating avoidable healthcare harm [4].

Particularly, patient care transitions from in-hospital to outpatient or at-home treatment are associated with high risks of adverse events, such as medication complications or nosocomial infections affecting approximately 20% of all inpatients [5–8]. Previous research showed that unplanned hospital readmissions and medication errors were more likely after poor care transitions [5, 9–12]. Moreover, patients reported better health status after effective care transitions [13–15]. One key to safe care transitions is good patient-centered communication between providers and patients during the discharge process [16, 17]. High quality discharge is characterized by different patient-centered actions, like including patients in their post-discharge care planning, supporting patients’ understanding of care needs and medications, and providing individual written care plans [13, 18, 19].

Although the research base on safe discharge practices is growing, there is still a need for a more profound and comprehensive understanding of the interplay between quality of care transitions, patient safety incidents, and patients’ health outcomes. The existing evidence is limited regarding external validity since it is primarily based on surveyed samples with restrictions on specific diseases or clinical specialties [15, 20], or patients’ characteristics like age and gender [13, 14, 21]. To the best of our knowledge, previous studies have mainly examined bivariate relationships, failing to account for the complexity of discharge processes. However, previous research indicated that the occurrence of safety incidents and patient’s health status at home after hospital discharge is affected by sociodemographic variables like age [22, 23] and gender [22–25]. Further, poorer health outcomes after discharge are related to treatment-related variables like the length of hospital stay [22, 23] and the need for intensive medical care, also referred to as the post-intensive care syndrome (PICS) [26]. Taken together, research shows that the quality of the discharge process has a significant influence on the occurrence of patient safety incidents and the health status of patients after discharge to home. In addition, sociodemographic and treatment-related variables were identified as relevant determinants of these outcomes after the discharge process. However, there is a lack of research to date in which the quality of care transition has been considered together with these relevant factors in one comprehensive approach. Therefore, it was our aim to consider these potential factors in one comprehensive model when examining the relationship between the quality of care transition and safety outcomes and health status. For this purpose, we applied a structural equation model (SEM) approach to investigate the complex interrelations in the discharge process and ensuing outcomes of care transitions from hospital to home. Our study thus contributes to the current evidence particularly in two ways:

First, by modeling the quality of care transition and potential outcomes in one comprehensive SEM, latent factors (versus manifest means) are included taking into account measurement errors and estimating robust, true construct variances [27]. This approach strengthens internal validity in investigating potential connections between care transitions and outcomes. This is particularly relevant because there is a need to understand how the complex discharge process affects various outcomes, such as adverse patient safety events at home and patients’ health status, taking into account other variables (e.g., patient sociodemographic and treatment characteristics). To validly represent these complex interactions, modeling all these variables in one comprehensive model, such as an SEM, is necessary [28].

Second, to overcome the limitations of previous studies regarding generalizability, data were obtained from various clinical areas and departments from patients with different diagnoses and diseases involved. Our study sample included patients from all departments of an academic teaching hospital (except pediatrics) to capture a heterogeneous sample in terms of patient and treatment characteristics. In addition, the sample sizes in many previous studies were small [13, 29, 30], so a large sample was generated in this study as a sound foundation for valid results. In addition to assessing health-related outcomes, we also included patient safety incidents after discharge to home in this study as patient-reported outcome measures (PROM). In order to strengthen external validity, we have furthermore used the Care Transitions Measure (CTM), which has been shown to be the most widely used patient-reported experience measure (PREM) to assess the quality of care transition [31].

To this end and to obtain a deep and comprehensive understanding of the complexity of discharge processes and ensuing safety and health-related outcomes, we established the following hypotheses. We examined the relationships between quality of care transition, patient safety incidents, and health outcomes under consideration of sociodemographic and treatment-related characteristics in one comprehensive multivariate analysis using an SEM approach:

- H1: Higher quality of care transition is associated with a lower likelihood of unplanned hospital readmissions,
- H2: Higher quality of care transition is associated with a lower likelihood of experiencing medication complications,
- H3: Higher quality of care transition is associated with better patient-reported health outcomes (physical and mental health status).

**Fig. 1:**
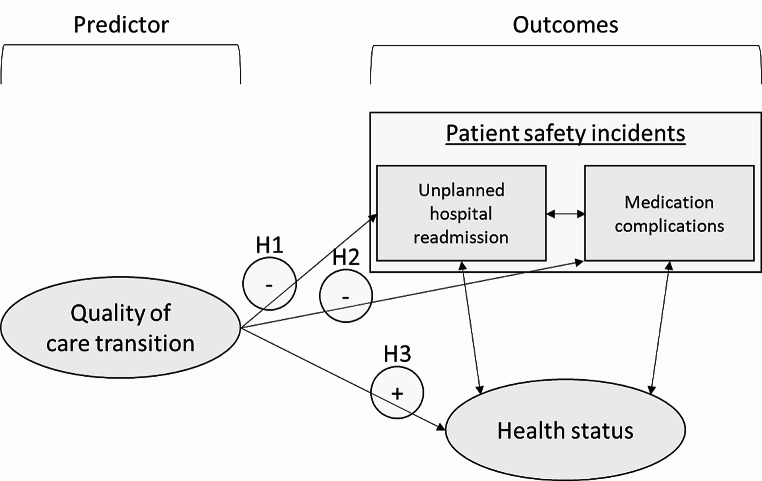
Conceptual model of quality of care transition during hospital discharge, patient safety, and health outcomes

Furthermore, we wanted to examine relationships between the occurrence of patient safety incidents and patients’ health status. Figure 1 shows our study’s conceptual model between the quality of care transition, patient safety outcomes, and patients’ health status.

## Methods

### Study setting and design

We conducted a hospital-wide cross-sectional study with patients surveyed after discharge from inpatient care. Data were collected via self-report questionnaires at an academic teaching hospital in Germany with maximum care in approximately 60 clinics and departments. The hospital provides care to around 350,000 outpatients and about 90,000 inpatients or emergency patients annually.

This study was conducted according to the Declaration of Helsinki. The Ethics Committee of the Medical Faculty of the University of Bonn approved the study protocol (Nr. 107/22). The study protocol was registered in the German Clinical Trials Register (DRKS; Nr. DRKS00028947). The study results are reported according to the Strengthening the Reporting of Observational Studies in Epidemiology guidelines [32].

### Participants

Inclusion criteria were patients at least 18 years old, discharged to home; inpatient treatment for at least three days. Consistently, patients from pediatric units were not included in this study. Altogether, 825 patients participated (response rate: 21%). Before analyses, we performed a data quality check. Ten cases were excluded as these patients indicated in a text field of the questionnaire that they were not at home after discharge. Further, we excluded eight questionnaires as they were returned later than two months. This was necessary to ensure that all patients interviewed reported about a similar period of time after hospitalization. The resulting final sample was 807 patient questionnaires. Of these, 666 (83%) patients completed the paper-pencil version and 141 via the online version. We performed a post hoc sample size calculation using WebPower [33]. Based on the finally tested model, we defined the following parameters: df = 198; effect size = 0.1; significance level = 0.05; and statistical power = 0.95. With these parameters, a sample size of *n* = 760 was needed to find significant effects. We could therefore assume that our sample size was sufficient.

### Data collection procedure

Patient addresses were generated via hospital administration records between May and August 2022. 3945 Patients received the paper-pencil questionnaire via mail about three weeks after hospital discharge. A stamped envelope was enclosed so that the questionnaire could be returned free of charge. Patients were given the opportunity to take part via an equivalent online version of the assessment via Unipark (Tivian XI GmbH), which was accessible via an URL or QR-Code printed in the paper-pencil questionnaire. There was no compensation for study participation. The assessment was designed to be anonymous, and participation was voluntary. The paper-pencil, as well as the online version of the assessment, included a comprehensive study disclosure and contact information for inquiries. In case patients were not able to participate, the questionnaire included the option to be filled in by relatives instead. Responses in both, online and paper-pencil version of the survey were continuously checked during the course of the study in order to recognize potential misunderstandings and ambiguities in answering the items. A free text field was provided at the end of the questionnaire for this purpose, but no misunderstandings were documented. The questionnaire data for the paper-pencil version were entered manually and randomly checked for correctness by the first author of the study.

### Measurements

#### Quality of care transition

The Care Transition Measure ([CTM] 19) was applied as the internationally most used assessment tool for quality of care transition [31]. A validation study regarding the German version of the CTM was previously conducted [34]. The questionnaire included 15 items measuring the quality of care transition on four dimensions (i.e., subscales): (a) *Critical understanding* assessed whether patients received an understanding of their own care responsibilities as well as insights into their medication and possible side effects (six items). (b) *Management preparations* represented the extent to which patients obtained an understanding of how to manage their health situation and knowledge about individual health-related red flags to which attention should be paid (four items). (c) *Care plan* measured whether patients received written plans of upcoming appointments and tests as well as plans regarding their own health care at home (2 items). (d) *Preferences important* was used to determine the extent to which patients and their caregivers were involved in decisions about their care after being discharged (3 items) [18, 19]. All items were answered on a 4-Point Likert Scale from 1 = ‘Strongly Disagree’ to 4 = ‘Strongly Agree’. A fifth response option was ‘Don’t Know/Don’t Remember/Not Applicable’, which was treated as missing values in the analyses.

#### Patient safety incidents and health assessment

As study outcomes, we assessed patient safety incidents as whether (1) patients experienced an unplanned need to attend a hospital or an emergency department, and (2) medication complications after their discharge from inpatient treatment by dichotomous items (no readmission / no medication complications, need of readmission / experience of medication complications). As there is no validated instrument for measuring patient safety incidents yet, these items were self-developed based on common adverse events after discharge [35] and have been successfully used in a previous study [36].

Further, we asked patients about their current physical (‘How do you rate your physical health (e.g., no physical limitations, pain)?’) and mental health (‘How do you rate your mental health (e.g., no feelings of anxiety, feeling depressed)?’) status on an 11-Point Likert Scale from 1 = ‘Very bad health’ to 11 = ‘Very good health’. These items were validly used in previous studies [37, 38].

#### Sociodemographic and treatment-related characteristics

Additionally, we assessed patients’ sociodemographic characteristics: age (in years) and gender (female, male, diverse). Further, treatment-related characteristics were assessed: length of hospital stay (in days); patients undergoing intensive medical care (yes, no).

The study items are presented in Appendix 1 in the Supplementary Material.

### Statistical analysis

In the first step, descriptive analyses of study variables were conducted. T-tests and bivariate correlations were used to examine potential group and subscale differences. Cronbach Alpha (CA) was used to estimate scale reliabilities of first-order factors, and McDonald’s Omega for second-order factors.

In the second step, we established an SEM on the proposed model shown in Fig. 1. Considering that we included categorical and numerical data in the modeling process, we used the robust weighted least square mean and variance adjusted (WLSMV) estimator for model estimation [39, 40]. To account for potential confounding variables, associations between sociodemographic, treatment-related characteristics, and study outcomes were examined prior to modeling. The SEM included characteristics with significant relations to study outcomes as covariates. The measurement model part of the SEM consisted of quality of care transition as constructed by the original authors’ proposed model of the CTM [19]: four subscales with a total of 15 items loaded on one common higher-level factor. Moreover, two items assessing the physical and mental health status constituted the latent factor of health status.

Within the structural part of the SEM, unplanned hospital readmissions, medication complications (patient safety incidents), and health status were regressed on the common factor of the quality of care transition. Moreover, we estimated the relationship between unplanned hospital readmissions and medication complications as well as the correlations between health status and both patient safety incidents.

Model parameters were evaluated using comparative fit index (CFI), Tucker Lewis index (TLI), root mean square error of approximation (RMSEA), root mean square residual (SRMR), and item factor loadings in consideration of the model fit criteria proposed by Hu & Bentler [41]. Therefore, the following criteria were considered to indicate good model fit: CFI ≥ 0.95, TLI ≥ 0.95, RMSEA ≤ 0.06, and SRMR ≤ 0.08. *P* < 0.05 was considered to be statistically significant in all analyses. Additional analyses aiming to gain a deeper understanding of CTM’s (sub-)scale effects on the study outcomes used logistic regression analyses. R and RStudio were used for data analyses [42, 43].

## Results

### Sample description

The study sample comprised 383 female patients (48%), 400 male patients (50%), and three persons who indicated their gender as diverse. Mean age was 60.8 years (SD = 18.0; Median = 64; Range = 19 to 94 years). Eighty questionnaires (68 paper-pencil, 12 online) were filled out by relatives (10%). Patients who used the online questionnaire were significantly younger than patients who returned paper-pencil questionnaires (M = 51.6 years vs. 62.6, respectively; t = 6.53, *p* < 0.001). On average, patients reported a duration of 10.1 days of inpatient stay in the hospital (SD = 16.3; Median = 5, Range 3 to 165 days). Of all patients, 206 (26%) received intensive medical care.

### Analyses for prevalence and associations with potential control variables

The CTM (range 1 to 4) had a mean score of 3.08 (SD = 0.77) and a reliability (McDonald’s Omega) of 0.93, indicating excellent internal consistency. CTM’s four subscales had the following means: preferences important: Mean = 3.09, SD = 0.94; management preparations: Mean = 3.15, SD = 0.86; care plan: Mean = 2.87, SD = 1.00; critical understanding: Mean = 3.09, SD = 0.79 with reliabilities of 0.92, 0.92, 0.68, and 0.91, respectively. All subscales reflect high levels of perceived quality of care transition. Yet, pairwise t-tests with adjusted p-values (holm-method) indicated that the CTM subscale *care plan* was significantly lower. Results are shown in Appendix 2 in the Supplementary Material.

Regarding patient safety incidents outcome, 71 patients (8.8%) reported unplanned hospital readmissions after initial discharge. Furthermore, 85 patients (10.5%) experienced medication complications after discharge. Of these, 19 (2.5%) patients experienced patient safety incidents and concurrent medication complications.

Regarding patients’ health status (range 1 to 11), the mean physical health was 6.97 (SD = 2.4), and the mean mental health was 7.61 (SD = 2.7).

We examined potential bivariate associations between sociodemographic characteristics and our study outcome variables and observed that higher age was significantly related to lower physical health (*r* = -0.25, *p* < 0.001). Additionally, higher mental health was associated with a shorter duration of hospital stay (*r* = -0.12, *p* < 0.01). Moreover, patients who underwent intensive medical care reported lower physical (t_718_ = 3.19, *p* < 0.01) and lower mental health (t_718_ = 2.32, *p* < 0.05). Patients’ gender was not associated with any study outcomes. Therefore, we included intensive medical care, age, and length of hospital stay as covariates in the following multivariate analyses. Age and length of hospital stay were mean-centered before modeling.

### Multivariate structural equation modeling – measurements characteristics

**Fig. 2:**
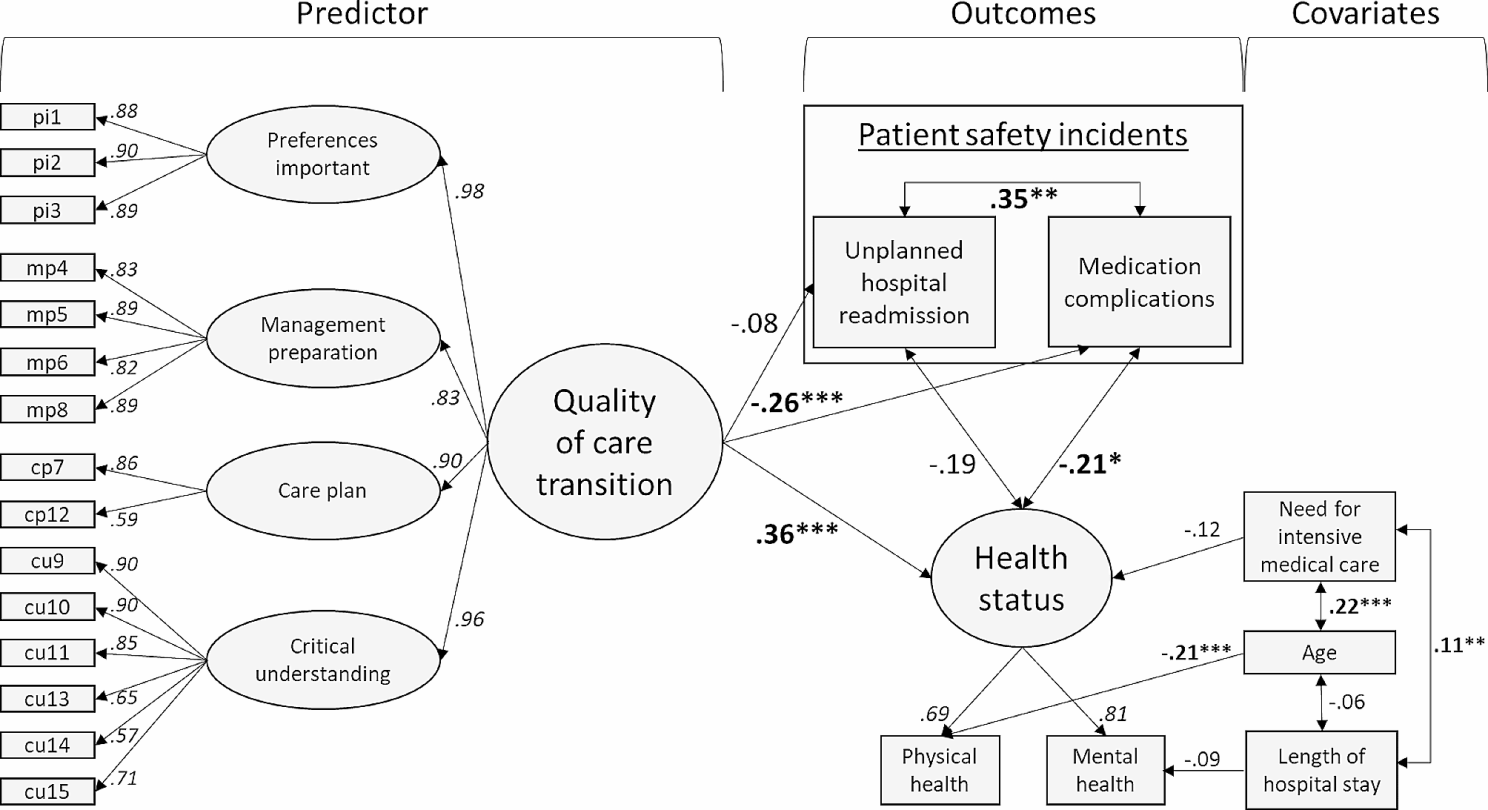
Structural equation model with standardized coefficients on quality of care transition, health status, and patient safety incidents. Italic numbers indicate factor loadings. Significant regressions and correlations are displayed in bold with * *p* < 0.05, ** *p* < 0.01, *** *p* < 0.001

To gain an understanding of the complex interrelations between the quality of care transition, patient safety incidents at home, and patients’ health status, we performed an SEM analysis under consideration of the above-mentioned covariates. Figure 2 shows the model and resulting estimates (without error terms).

As shown in Fig. 2, the quality of care transition was validly assessed by four subscales: *preferences important, management preparation*, *care plan*, and *critical understanding*. All items had high significant factor loadings (all λ >= 0.57) on their respective subscale, corroborating the factorial validity of the CTM tool. The patients’ health status was validly measured (all factor loadings λ >= 0.69) by two items assessing physical and mental health with an acceptable reliability of 0.73. Model fit parameters were good with CFI = 0.937, TLI = 0.926, RMSEA = 0.032, and SRMR = 0.060.

### Multivariate associations and hypotheses testing

Concerning our first hypothesis, we found that the likelihood of unplanned hospital readmissions was unrelated to the overall quality of care transition (B = -0.01, β = -0.08, *p* = 0.39). Consequently, our first hypothesis was rejected.

Our second hypothesis proposed that higher quality of care transition is associated with a lower likelihood of medication complications. Hypothesis 2 was empirically confirmed by our model: B = -0.35, β = -0.26, *p* < 0.01.

Concerning our Hypothesis 3, a high, positive association between quality of care transition and patients’ overall health status was observed (B = 0.74, β = 0.36, *p* < 0.001).

Furthermore, lower health status was significantly related to increased reports of medication complications (*r* = -0.21, *p* < 0.05), but not with a lower likelihood of unplanned hospital readmissions (*r* = -0.19, *p* = 0.08). After multivariate adjustment, we identified a significant association between the likelihood of medication complications and unplanned hospital readmissions (*r* = 0.35, *p* < 0.01).

### Additional analyses on CTM subscales and study outcomes

To better understand which aspects of the care transition (i.e., respective CTM subscale) contributed to improved health status and a lower likelihood of medication complications, we subsequently established another SEM with CTM subscales as individual predictors. Covariates age, intensive medical care, and length of stay were consistently used as in the previous model (cf., Fig. 2). In this expanded model, none of the four CTM subscales significantly predicted the occurrence of medication complications or patients’ health status. Since we further assumed that a positive effect of quality of care transition only occurs when all aspects (subscales) of a safe discharge process act together, we used, moreover, regression analyses to estimate associations between CTM’s total mean score and our study outcomes. The likelihood of medication complications was significantly predicted by the CTM total mean score: B = − 0.67, Odds ratio = 0.51 (95% confidence interval [CI]: 0.39–0.68), *p* < 0.001. Likewise, the health status depended on the CTM total mean score (B = 0.84 [CI: 0.64–1.04], β = 0.28, *p* < 0.001).

## Discussion

### Principal findings

Although patient’s discharge processes have been acknowledged as susceptible to risks and potential harm, establishing and safeguarding transitional safety remains a significant challenge in complex care systems [44]. Improving patient transition processes between different care contexts is a fundamental pillar of safe patient care [45]. Our study aimed to gain a deeper understanding of the complex interrelations between the quality of discharge processes, the likelihood of patient safety incidents at home, and the patients’ health status in one comprehensive model. Our findings contribute to existing knowledge by using a multivariate SEM approach, spanning various medical specialties within a large sample size, and considering sociodemographic and treatment-related characteristics as covariates. Moreover, by using the standardized CTM as a PREM, we advance our understanding of how patients appraise various process steps of care in the course of hospital discharge. Further, we applied PROMs to investigate patient safety incidents and patients’ health status [46]. PREMs and PROMs are key drivers of patient-centered care, and this study contributes significantly to the current research base of patients’ perspectives about the care process and outcomes by applying both patient-reported performance measures in one model [46, 47].

Regarding our first hypothesis, we expected that higher quality in care transition is associated with fewer unplanned hospital readmissions. Our empirical data did not support this assumption. However, this resonates well with previous research that is somewhat inconsistent with a heterogonous base of studies that did not find a connection between the quality of care transition and readmissions [20, 48, 49]. Post-hoc, a potential explanation could be that previous research did not consider the complex interactions of different patient safety incidents, health-related aspects, as well as sociodemographic and treatment-related characteristics. We assume that the probability of re-hospitalizations could be an outcome of medication complications rather than a direct outcome of the quality of discharge processes. This assumption is corroborated by the significant association found in our data between readmissions and medication complications, as well as previous research highlighting that medication-targeted discharge interventions are associated with fewer readmissions [50].

Referring to the second hypothesis, our analysis confirmed a significant association between higher quality in care transition processes and a lower likelihood of medication complications. This result underscores the importance of the WHO Global Patient Safety Challenge ‘Medication Without Harm’ [51] and the particular need to take medication safety in care transitions into account [52]. Previous research has shown that about 50% of patients experience medication complications (e.g., errors or unintentional medication discrepancies) after hospital discharge [8]. Most medication complications after discharge occur due to lacks in transitional care [53]. Our study thus confirms that medication safety in the discharge process plays an influential role in patient safety. Various studies have shown that interventions to improve medication safety in the discharge process, e.g., patient education, medication reconciliation, and improved communication between healthcare professionals reduce medication complications [54–57]. Our study contributes to a better understanding of the complexity and possible impact of medication complications in the context of poor quality of care transitions: medication complications are associated with a higher probability of readmission as well as with poorer patients’ health status. This finding corroborates that medication complications are probably not only the outcome of poor quality of care transition but are likely to lead to more readmissions and poorer patient health [57]. Future research should thus explore these possible mediation effects of medication complications in longitudinal studies.

Our third hypothesis expected a positive relationship between quality in care transitions and patients’ health status. Besides patients’ age, we also considered treatment-related characteristics as potential covariates and found a significantly positive relationship between the quality of care transition and patients’ health status. This finding is consistent with previous research [13–15]. Interestingly, however, there was no correlation between health status and readmissions, which indicates that patients rate their health worse if the quality of care transition was experienced as poor, regardless of the need for readmissions. Due to the differential association between quality of care transition, medication complications, and health-related outcomes, we assume that poor quality of care transition may have a two-fold effect: it leads to poorer health in some patients and medication complications in others. This elucidates that studies that only record patient safety incidents as outcomes overlook that many people suffer from health constraints due to poor quality of care transitions without experiencing medication complications or readmissions. This underpins the assumption that discharge is a complex process that can be associated with individual outcomes for patients and therefore requires discharge processes in which the unique needs of the patients are considered [58].

Our subsequent analyses emphasized that single aspects of safe care transitions (i.e., CTM subscales) are not predictive of patient safety nor patients’ health, but the CTM total mean score was. Our findings suggest that safe care transitions are not merely a question of single actions but a bundle of several interacting and complementary actions that collectively foster safety and patients’ health after discharge from the hospital to home [59]. This observation provides further evidence for designing improvement approaches such that multi-component programs addressing various challenges of the discharge process are more effective in reducing patient safety incidents [60].

Summarizing, the results of this study implicate that discharge is a complex process in which various sociodemographic and treatment-related characteristics impact patient outcomes. This highlights the need for future research to consider this complexity for making valid statements about this vulnerable care process, which is characterized by interactions of different systems, care, and patient influences. To the best of our knowledge, this study is the first to demonstrate the complexity of the discharge process and the interactions of different characteristics in studying patient experiences and outcomes in one comprehensive model. Our study findings contribute significantly to important gaps in previous research: firstly, the results of this study go beyond a mere consideration of bivariate relationships by comprehensively considering the multivariate nature and interplay of potentially influencing factors in one comprehensive SEM. Secondly, our research approach drawing upon a large and heterogeneous sample in terms of the specialties, age groups, and diagnostics, eventually enabled us to establish a high level of external validity. Our findings may inform future investigations such to not simplify the complexity of discharge processes using bivariate analyses but to account for the interactions of different characteristics, patient experiences, and patient outcomes through multivariate modeling. Furthermore, our study has shown that sociodemographic and treatment-related factors determine patient outcomes. Thus, our results support calls from other research for patient-centered and individualized discharge planning to ensure transitional patient safety [58].

### Limitations

The results of our analyses must be interpreted in consideration of several limitations. We used a convenience sampling approach for data collection which may introduce a sampling bias into our data. In addition, we conducted a cross-sectional study which does not allow for causal inferences. As half of the study participants were older than 64 years, our findings may be limited to elderly patients and are likely to be not fully representative of the needs of younger patients. Another limitation is that this study used self-reported data from patients only. Nonetheless, it has been shown that patients can provide valid information about adverse events [61]. We collected data from patients treated in one academic hospital in Germany, which may limit the external validity of our results to other healthcare contexts and systems. Therefore, our findings should be replicated in multi-center studies as well as in the health systems of other countries. A further limitation is that the patient safety outcomes were assessed using single items. As no internationally validated instrument for measuring these relevant patient safety incidents is yet available, future research should take this important gap into account. Further, mixed methods studies or process evaluations via expert observations, provider reports, or similar methods might provide a more nuanced picture concerning the varying challenges in the course of hospital discharge. Lastly, although we sought to include a relevant set of sociodemographic and treatment-related characteristics to account for potential confounding influences, we acknowledge that our selection may be limited and further determinant factors may be influential.

## Conclusion

High quality of care transition and patient-centeredness during discharge processes are associated with a lower likelihood of medication complications and better patients’ health status at home. These effects bear up against the influences of measurement errors and sociodemographic and treatment-related characteristics in a comprehensive SEM. Quality of care transition was not related to the likelihood of unplanned hospital readmission in our multivariate model. Our analyses propose that it is unlikely that single aspects of high quality care transitions alone yield positive effects in terms of patient safety and health-related outcomes. Moreover, the discharge process should be designed in a patient-centered and safe manner through careful consideration and composition of several complementary actions and patient-oriented measures.

### Electronic supplementary material

Below is the link to the electronic supplementary material.

Supplementary Material 1

## Data Availability

The datasets used and/or analyzed during the current study are available from the corresponding author on reasonable request. Please contact the corresponding author Matthias Marsall (matthias.marsall@ukbonn.de).

## Abbreviations

DRKS: German Clinical Trials Register (DRKS)
CA: Cronbach’s alpha
CFI: comparative fit index
CI: confidence interval
CTM: care transitions measure
RMSEA: root mean square error of approximation
SEM: structural equation model
SRMR: root mean square residual
TLI: Tucker Lewis index
WLSMV: weighted least square mean and variance adjusted
