# Supplementary material for: Quality of care transition, patient safety incidents, and patients’ health status: a structural equation model on the complexity of the discharge process
Source: BMC Health Serv Res. 2024 May 3;24:576. doi: 10.1186/s12913-024-11047-3 (PMC11069201; doi:10.1186/s12913-024-11047-3)
Supplement: Supplementary file 1 — Supplementary Material 1 [file 12913_2024_11047_MOESM1_ESM.docx]

**Appendix 1: Study items**

Quality of care transition (CTM; © Eric A. Coleman, MD, MPH all rights reserved)

Response options: 1 = Strongly Disagree - 4 = Strongly Agree; 5 = Don't Know/Don't Remember/Not Applicable

| **#** | **Item (Original)** | **German item** |
| --- | --- | --- |
| 1 | Before I left the hospital, the staff and I agreed about clear health goals for me and how these would be reached. | Vor der Entlassung aus der Klinik einigten sich das Klinikpersonal und ich auf Ziele für meine Gesundheit und wie ich diese erreichen kann. |
| 2 | The hospital staff took my preferences and those of my family or caregiver into account in deciding what my health care needs would be when I left the hospital. | Das Klinikpersonal berücksichtigte meine Vorstellungen und die meiner Angehörigen bei der Entscheidung, was meine gesundheitlichen Bedürfnisse nach der Entlassung aus der Klinik sind. |
| 3 | The hospital staff took my preferences and those of my family or caregiver into account in deciding where my health care needs would be met when I left the hospital. | Das Klinikpersonal berücksichtigte meine Vorstellungen und die meiner Angehörigen bei der Entscheidung, wo (z.B. zu Hause, ambulante Versorgungseinrichtung) meine gesundheitlichen Bedürfnisse nach der Entlassung aus der Klinik am besten erfüllt werden. |
| 4 | When I left the hospital, I had all the information I needed to be able to take care of myself. | Bei der Entlassung aus der Klinik hatte ich alle notwendigen Informationen, um mich selbst versorgen zu können. |
| 5 | When I left the hospital, I clearly understood how to manage my health. | Bei der Entlassung aus der Klinik hatte ich genau verstanden, wie ich mit meiner Gesundheit umgehen muss. |
| 6 | When I left the hospital, I clearly understood the warning signs and symptoms I should watch for to monitor my health condition. | Bei der Entlassung aus der Klinik hatte ich genau verstanden, auf welche Warnzeichen und Symptome ich achten muss, um meinen Gesundheitszustand zu überwachen. |
| 7 | When I left the hospital, I had a readable and easily understood written plan that described how all of my health care needs were going to be met. | Bei der Entlassung aus der Klinik habe ich schriftliche und leicht verständliche Informationen darüber erhalten, wie meine gesundheitliche Versorgung sichergestellt werden kann. |
| 8 | When I left the hospital, I had a good understanding of my health condition and what makes it better or worse. | Bei der Entlassung aus der Klinik hatte ich ein gutes Verständnis für meinen Gesundheitszustand und was diesen verbessert oder verschlechtert. |
| 9 | When I left the hospital, I had a good understanding of the things I was responsible for in managing my health. | Bei der Entlassung aus der Klinik hatte ich ein gutes Verständnis für die Dinge, um die ich mich hinsichtlich meiner Gesundheitsversorgung selbst kümmern muss. |
| 10 | When I left the hospital, I was confident that I knew what to do to manage my health. | Bei der Entlassung aus der Klinik wusste ich genau, was ich für meine Gesundheit tun muss. |
| 11 | When I left the hospital, I was confident I could actually do the things I needed to do to take care of my health. | Bei der Entlassung aus der Klinik war ich überzeugt, dass ich tatsächlich das tun kann, was ich für meine Gesundheit tun muss. |
| 12 | When I left the hospital, I had a readable and easily understood written list of the appointments or tests I needed to complete within the next several weeks. | Bei der Entlassung aus der Klinik habe ich eine schriftliche und leicht verständliche Liste mit Terminen oder Untersuchungen für die nächsten Wochen erhalten. |
| 13 | When I left the hospital, I clearly understood the purpose for taking each of my medications. | Bei der Entlassung aus der Klinik hatte ich genau verstanden, wofür ich jedes meiner Medikamente einnehmen sollte. |
| 14 | When I left the hospital, I clearly understood how to take each of my medications, including how much I should take and when. | Bei der Entlassung aus der Klinik hatte ich genau verstanden, wie ich jedes meiner Medikamente einnehmen muss (inklusive Dosierung und Zeitpunkt der Einnahme). |
| 15 | When I left the hospital, I clearly understood the possible side effects of each of my medications. | Bei der Entlassung aus der Klinik hatte ich genau verstanden, welche möglichen Nebenwirkungen jedes meiner Medikamente haben kann. |

**Patient Safety Incidents**

Response options: No vs. Yes

| **Variable** | **Item** |
| --- | --- |
| unplanned hospital readmissions | Did you need to be readmitted to a hospital/emergency room unplanned after your discharge from the hospital? |
| medication complications | Have you had any difficulties with taking your medication (e.g. wrong dosage; unexpected side effects, etc.)? |

**Health Assessment**

Response options: 1 = Very bad health - 11 = Very good health

| **Variable** | **Item** |
| --- | --- |
| Physical health | How do you rate your physical health (e.g., no physical limitations, pain)? |
| Mental health | How do you rate your mental health (e.g., no feelings of anxiety, feeling depressed)? |

**Sociodemographic and treatment-related characteristics**

| **Variable** | **Item** | **Response options** |
| --- | --- | --- |
| Age | Please enter your age: | Text box (years) |
| Gender | Please enter your gender: | 1 = Female  2 = Male  3 = Diverse |
| Length of hospital stay | How long were you hospitalized during your last stay at the clinic? | Text box (days) |
| Need for intensive medical care | Did you receive intensive medical care during your last stay at the clinic? | 1 = No  2 = Yes |

**Appendix 2: Mean differences of the Care-Transitions Measure subscales**
